# Innovative Processing Technologies for Developing Functional Ingredients and Food Products with Health Benefits from Grains

**DOI:** 10.3390/foods12071356

**Published:** 2023-03-23

**Authors:** Cristina Martínez-Villaluenga, Elena Peñas

**Affiliations:** Institute of Food Science, Technology and Nutrition (ICTAN-CSIC), José Antonio Novais 6, 28040 Madrid, Spain

Grains are dry seeds belonging to diverse crops, including cereals, pseudocereals and pulses. They have been part of the human diet since ancient times because, in addition to being one of the most important sources of protein and carbohydrates in human diets, they contain high amounts of other nutrients, such as fibre, minerals and vitamins. Grains also contain a wide range of unique bioactive compounds (β-glucans, phenolics, carotenoids, tocotrienols, among others) that are linked with the prevention of chronic diseases, including cancer, cardiovascular diseases and type 2 diabetes [1,2].

Feeding the ever-growing world population while avoiding environmental damage brings new challenges and opportunities to the food industry and agriculture sectors. Grains can be important contributors for developing a more resilient, nutritious and sustainable food supply chain. In fact, the development of grain-derived foods is in agreement with consumer demand for healthier and more sustainable food options. The conventional food processing technologies involve mechanical, chemical and physical transformations that may have a negative impact on the content, bioavailability and biological activity of bioactive compounds present in grains, thus reducing the health benefits of the resultant grain-derived foods. This fact, together with the need to stimulate the transition towards a circular economy model, promote research regarding innovative and eco-friendly processing technologies that retain the nutritional and functional quality, the freshness and sensory attributes of food products. Technologies, such as germination, fermentation and enzymatic hydrolysis are among the most promising technologies used in recent years to achieve these purposes.

This Special Issue comprises 11 selected original articles and 1 review covering a wide range of studies regarding the application of innovative technological approaches to enhance the nutritional and bioactive potential of ingredients and foods derived from grains.

Germination is a physiological process of emerging plants from seeds that produces the hydrolysis of matrix-bound phytochemical compounds and the formation of new bioactive compounds. Therefore, it represents an attractive technological strategy to enhance the bioaccessibility and accumulation of bioactive compounds in grains. A part of this issue is, in fact, devoted to improvements in nutritional and nutraceutical properties of pulses, cereals and other grains. In this regard, Rico et al. [3] optimized germination conditions to produce lentil flours with an improved nutritional and bioactive profile. The study investigated the influence of temperatures in a range of 15–27 °C and periods of 1–5 days on the content of different nutrients (proximate composition, ascorbic acid, fatty acids), antinutrients (phytic acid) and bioactive compounds (γ-aminobutyric acid, phenolic compounds), as well as on antioxidant activity, expected glycemic index and colour of the sprouted lentil flours. Applying response surface methodology (RSM), the authors demonstrated that germination caused the accumulation of ascorbic acid, γ-aminobutyric acid and insoluble phenolic compounds and increased the antioxidant activity and expected glycemic index of flours, while reducing the content of phytic acid and modifying the colour or the germinated lentil flours. The study identified 21 °C and 3.5 days as the most suitable germination conditions to produce sprouted lentil flours with maximized quality. Similarly, the influence of germination conditions on nutritional and health-promoting compounds of moringa seeds were explored by Coello et al. [4]. Sprouted moringa seeds showed higher levels of protein, fat, fibre, riboflavin, γ-aminobutyric, phenolic compounds, some individual glucosinolates and antioxidant activity than non-germinated seeds. A multiresponse optimization of germination conditions was performed to identify 28 °C and 24 h as the optimal conditions to enhance the accumulation of riboflavin, phenolics and antioxidant activity of germinated moringa seed. Moreover, 36 °C and 24 and 96 h were the most suitable germination conditions to improve the glucosinolates and γ-aminobutyric acid levels and thiamine content, respectively, in sprouted moringa. This study shows that sprouted moringa is an interesting ingredient for developing innovative plant-based functional foods. Along the same line, Fukushima et al. [5] provided evidence for reductions in phytic acid content in brown rice by increasing the temperature of the soaking step during germination. The results of the study revealed that the application of 50 °C during brown rice soaking enhanced the activity of phytase enzyme, thus notably reducing the levels of phytic acid in germinated brown rice grains. However, soaking temperature did not affect the zinc (Zn) content and improved the calculated total daily absorbed Zn in germinated brown rice. The authors of the study suggested that an increase in temperature above 50 °C during grain imbibition is a valuable approach to improve the bioavailability of Zn in germinated brown rice. Another study conducted by León-López et al. [6] reported the enormous potential of elicitation to improve the content of bioactive compounds and the health-promoting properties of legume sprouts. The study focused on the application of response surface methodology (RSM) to optimize the concentration of the hydrogen peroxide (H_2_O_2_) elicitor and germination time to maximize the content of total phenolics and flavonoids as well as the antioxidant activity of chickpea sprouts. The authors demonstrated that chickpea sprouts with optimum features can be produced by using 30 mM of H_2_O_2_ and a germination time of 72 h. The sprouts obtained in these conditions showed the highest levels of total phenolics, flavonoids and antioxidant activity and the lowest content of phytic acid and saponins. This study supports the application of elicitation as an effective strategy to improve the bioactive potential of sprouted chickpea.

Enzymatic hydrolysis has proven to be a valuable tool for enhancing the bioaccessibility and health-promoting properties of ingredients and by-products from grains. Particularly, in the study of Villanueva-Lazo et al. [7], the aim was to obtain a functional ingredient from chia protein isolate through enzymatic hydrolysis by Alcalase and Flavourzyme. High-quality protein hydrolysates were produced by both enzymes, although Alcalase treatment for 15 min (CPH15A) led to a higher degree of hydrolysis, antihypertensive and antioxidant properties. Moreover, compared to chia defatted flour and protein isolate, techno-functional properties of CPH15A showed improved emulsifying and stabilizing properties. This study revealed that CPH15A has potent antihypertensive and antioxidant properties and can constitute an effective alternative to other plant protein ingredient sources that are being used in the food industry. Saponin-rich extracts from edible seeds have gained increasing interest and their hydrolysis may be an effective strategy to enhance their potential bioactivity. Navarro del Hierro et al. [8] studied the resulting chemical modifications of fenugreek and quinoa saponin-rich extracts after hydrolysis as well as their impact on the subsequent bioaccessibility of bioactive compounds. After in vitro digestion, non-hydrolyzed saponins from fenugreek and quinoa extracts were fully bioaccessible, sapogenins from hydrolyzed fenugreek extracts displayed a good bioaccessibility (76%) and the sapogenin from hydrolysed quinoa extracts was moderately bioaccessible (38%). Digestion of saponin and sapogenin standards suggested that other components of the extracts were enhancing the bioaccessibility. Other minor bioactive compounds (phytosterols, alkylresorcinols, tocols and some phenolics) also displayed optimal bioaccessibility values (70–100%). Recycling by-products from the food industry has become a central part of research to help create a more sustainable future. To valorise wheat and rye bran, Juhnevica-Radenkova et al. [9] designed a biorefining process accomplished by non-starch polysaccharide-degrading enzymes for the release of ferulic acid (FA) for food and pharmaceutical applications. Up to 11.3 and 8.6 g kg^−1^ of FA was released from rye and wheat bran, respectively, upon 24 h enzymatic hydrolysis with multi-enzyme complex Viscozyme^®^ L. This approach is an environmentally friendly alternative with a safer profile than conventional processing and could represent the future for sustainable industrial-scale production of ferulic acid.

Recently, there has been renewed interest in fermentation as it presents opportunities for improving the nutritional and functional properties of food. Fermentation is a promising tool to boost innovation in the plant-based beverage sector as it may provide several advantages in fermented products, including high nutritional value, pleasant palatability and potential healthy properties. In the framework of this research topic, Cardinali et al. [10] tested a pool of 23 lactic acid bacteria strains as monocultures for the fermentation of three ad hoc formulated cereal- (red rice and barley) and pseudocereal (buckwheat)-based substrates. Eight strains with the best performance in terms of acidification rate were selected for the formulation of three multiple strain cultures to be further exploited for the manufacture of laboratory-scale prototypes of fermented beverages. The composition and microbiological features of the three experimental beverages highlighted their high biological value for further exploitation. In another study, Aparicio-Garcia et al. [11] investigated the use of sprouted oat flour as a substrate to develop a novel gluten-free fermented beverage using a probiotic (*Lactobacillus plantarum* WCFS1) starter culture. Physicochemical, microbiological, nutritional and sensory properties of sprouted oat fermented beverage (SOFB) were characterized. SOFB was a good source of proteins, β-glucans, thiamine, riboflavin and phenolic compounds, and had a high antioxidant potential. Spoilage and pathogenic microorganisms were not detected in SOFB. In a sensory evaluation analysis, SOFB was well accepted by panellists. Viability of *L. plantarum* and β-glucans content was stable during SOFB shelf-life period. SOFB fulfils current consumer demands regarding natural and wholesome plant-based foods. Fermentation also represents a promising tool to valorise food by-products, improving ingredient functionality and technological quality. Brewers’ spent grain is one of the main side-streams of the brewing industry, rich in protein and fibre. Neylon et al. [12] fortified wheat bread with spray-dried brewers’ spent grain (BSG) and fermented brewers’ spent grain (FBSG) at two addition levels to achieve “source of fibre” and “high in fibre” claims according to EU regulations. The inclusion of BSG and FBSG resulted in a stronger and faster gluten development, reduced starch-pasting capacity, and increased dough resistance/stiffness. However, fermentation improved bread characteristics resulting in increased specific volume, reduced crumb hardness, and restricted microbial growth rate over time. Additionally, the inclusion of FBSG slowed the release in reducing sugars over time during in vitro starch digestion. Thus, fermentation of BSG can ameliorate bread techno-functional properties and improve nutritional quality of breads.

The emerging interest of consumers in foods having high nutritional value and beneficial health implications has resulted in a remarkable boost of probiotic and prebiotic food product development. Cereals, as one of the main components in the human diet, contain substantial levels of dietary fibre with probable prebiotic potential. In addition, dietary fibre, particularly soluble dietary fibre, has recently emerged as a promising natural highly functional food ingredient in food production. Abdi and Joye [13] reviewed the prebiotic potential of cereal dietary fiber types and covered the achievements and developments regarding their isolation. Different components of dietary fibre and their effect on the host bacteria through in vitro and/or in vivo studies are reviewed. Furthermore, means of boosting the prebiotic properties of cereal components and innovative strategies for the extraction of cereal dietary fibre are discussed.

The occurrence of recalls involving pathogenic *Escherichia coli*-contaminated wheat flours shows the need for incorporating antimicrobial interventions in wheat milling. Rivera et al. [14] assessed the efficacy of sodium bisulfate (SBS) tempering in reducing *E. coli* O121 (ATCC 2219) and O26 (ATCC 2196) wheat load and to evaluate the impact of effective (≥3.0 log reductions) SBS treatments on wheat flour quality. The addition of SBS during tempering resulted in *E. coli* (O121 and O26) log reductions. SBS tempering produced acidic wheat flours but had comparable wheat flour properties in terms of composition, dough and bread-making properties relative to the control.
